# A Case Report of Lifesaving Intravenous Bolus Epinephrine Administration in a Case of Severe Refractory Anaphylactic Shock

**DOI:** 10.7759/cureus.28249

**Published:** 2022-08-22

**Authors:** Mustafa Hammad, Noora Adam, Khurram Sarfaraz, Maryam Adam, Naser Mansoor

**Affiliations:** 1 Medicine, Salmaniya Medical Complex, Manama, BHR; 2 Department of Emergency Medicine, Salmaniya Medical Complex, Manama, BHR; 3 Department of Internal Medicine, Salmaniya Medical Complex, Manama, BHR

**Keywords:** life-threatening anaphylaxis, allergy, clinical case report, anaphylactic shock, bolus intravenous epinephrine

## Abstract

Anaphylaxis is a life-threatening response to various types of allergens. Early recognition and management are crucial for reducing mortality. This case report highlights a 31-year-old male with a background of hypertension who presented to the emergency department with nausea, vomiting, right flank pain, headache, and elevated blood pressure (BP) of 212/134 mmHg. The patient was started on stat captopril 12.5 mg tablet and stat amlodipine 5 mg tablet for his high BP and stat diclofenac 75 mg (1 mg/kg) intramuscular (IM) for his flank pain. Two minutes later the patient started developing swelling of his mucosal membranes with no urticaria or rashes and his BP suddenly dropped and was unrecordable. First-line management was immediately initiated including the administration of two standard adult doses of IM epinephrine of 500 mcg each with a 5-minute interval. The BP remained undetectable; accordingly, a third IM epinephrine dose of 500 mcg was administered along with an intravenous (IV) epinephrine drip initiated at a rate of 4 mcg/min. The BP became 60/40 mmHg but kept dropping, thus an IV epinephrine bolus of 300 mcg (4 mcg/kg) was given along with the ongoing IV epinephrine drip. BP increased to 126/75 mmHg. While on the IV epinephrine drip the BP dropped again to 88/59 mmHg, a second IV epinephrine bolus of 200 mcg (2.6 mcg/kg) was given and the BP became 140/90 mmHg and recovery was achieved. Emergency cases require immediate recognition and intervention. Currently, IM epinephrine is the primary treatment for anaphylaxis. We hope our case report contributes to the database on severe refractory anaphylaxis by discussing a successful case where IV bolus epinephrine was used to prevent imminent cardiovascular collapse. Highlighting the need for appropriate escalation of management given the availability of physicians with expertise.

## Introduction

Anaphylaxis is an acute hypersensitivity reaction to a stimulus that can be life-threatening if early measures are not taken promptly [1]. The prevalence of anaphylaxis is 0.05-2% and 3% in the United States of America and Europe, respectively [1]. It can be triggered by various factors such as food, medications, insect stings, and many other agents [1]. Patients can exhibit various systematic signs and symptoms that can range from mild reactions with skin manifestations to severe life-threatening reactions such as respiratory compromise and hypotension [1].

Early recognition of anaphylaxis clinical presentation and early medical intervention is vital in reducing the chances of developing anaphylactic shock [2]. Reversing anaphylaxis can be achieved by removing the causative agent if possible, assessing the airway, breathing, circulation, and injecting intramuscular (IM) epinephrine repeatedly as needed [3].

In this report, we would like to emphasize the importance of rapid and early stabilization of patients with anaphylaxis and highlight our approach to overcoming and successfully managing a case of severe anaphylactic shock refractory to standard first-line therapy.

## Case presentation

A 31-year-old Pakistani male with a known case of hypertension for 2 years presented to the emergency department complaining of nausea, vomiting, right flank pain, and headache. The patient reports non-compliance with his therapeutic regimen as he is uninsured and cannot afford his medications.

The right flank pain started one day ago and was associated with nausea and three episodes of vomiting. The vomitus was of normal food content, non-bloody, non-projectile, and non-bilious. The patient has a one-month history of recurrent moderate, intermittent right flank pain radiating to the right groin worsened with urination and is not associated with fever or rigors. Also, the headache started in the last 2 months, worsening in the last week and it was described as a generalized headache, moderate in severity, and not associated with changes in vision or photophobia. The patient is not following up with his general practitioner and is not currently taking any medications, has not had any previous surgeries, has no known allergies, and does not report any insect bites. He works an office job and does not smoke or drink alcohol. Family history is positive for hypertension on the paternal side.

On examination, the patient looked well, alert, oriented, and not in distress. The patient had mild tenderness on head palpation and over the right flank. His vital signs were as follows: temperature 37°C, blood pressure (BP) 212/134 mmHg, heart rate 78 beats/minute, respiratory rate 12 breaths/minute, and oxygen saturation 100% on room air. The patient’s weight and height were 75 kg and 1.7 m, respectively with a calculated body mass index of 25.95 kg/m^2^.

The patient was triaged to level 4 care and was started on a stat dose of captopril 12.5 mg tablet. His BP lowered to 191/116 mmHg over a period of 1 hour. A stat dose of amlodipine 5 mg tablet was given to help lower his BP further along with a stat dose of diclofenac 75 mg (1 mg/kg) intramuscular (IM) for his flank pain. Two minutes later, the patient started getting agitated and the nurses noticed that the patient started developing swelling of his mucosal membranes, no urticaria, or rashes were visualized and his BP suddenly dropped and became unrecordable. The patient was immediately given two standard adult doses of IM epinephrine injections for anaphylaxis into the right deltoid of 500 micrograms each with a 5-minute interval (0.5 mL of 1 mg/mL, 1:1000 epinephrine), two large-bore cannulas started and 1 L of normal saline (NS) was administered, the patient was placed on high-flow oxygen, stat IV hydrocortisone 300 mg (4 mg/kg), stat IV promethazine 25 mg (0.33 mg/kg), and albuterol nebulization of 1 mL over 5 minutes was given three times back-to-back. However, his BP did not pick up. The patient was immediately shifted to level 2 care where he was connected to a cardiac monitor and a continuous pulse oximeter and given a third IM epinephrine injection of 500 mcg. An IV epinephrine drip was started at a rate of 4 drops/min (1 mg of epinephrine 1:1000 in 1 L NS i.e., 4 mcg/min). The patient’s BP reached 60/40 mmHg. However, it was not maintained and was progressively dropping down. A decision by the team leader was to administer an IV epinephrine bolus of 300 mcg (3 mL stat of (1 mL of 1/10,000 epinephrine + 9 mL of NS) i.e., 4 mcg/kg). The patient’s BP raised to 126/75. After 10 minutes, the BP dropped again to 88/59 mmHg, hence giving another IV epinephrine bolus of 200 mcg (2 mL stat of (1 mL of 1/10,000 epinephrine + 9 mL of NS) i.e., 2.6 mcg/kg). His BP picked up to 140/90 mmHg. The patient started shivering and his lips and nose swelling started to resolve. Ear-nose and throat and intensive care unit on-call doctors were consulted immediately following the recognition of this anaphylaxis. No airway obstruction was visualized and intubation was not necessary. A summary of the timeline of events that happened during this case is summarized in Table 1 and the laboratory investigations following the anaphylactic episode are summarized in Table 2.

**Table 1:** Timeline demonstrating the interventions and corresponding vitals of the patient following administering tab amlodipine and IM diclofenac. IM: intramuscular; IV: intravenous; BP: blood pressure; HR: heart rate; BPM: beats per minute; O2: oxygen Tab captopril was administered 1 hour before administering tab amlodipine and IM diclofenac.

Time of administering medications	Intervention	Vitals
0 minutes	Tab amlodipine 5 mg + IM diclofenac 75 mg (1 mg/kg)	BP 191/116 mmHg, HR 76 BPM, O_2_ saturation 100%
2 minutes	First IM epinephrine dose 500 micrograms (standard adult dose)	BP unrecordable, HR 102 BPM, O_2_ saturation 88%
7 minutes	Second IM epinephrine dose 500 mcg (standard adult dose) + stat IV hydrocortisone 300 mg (4 mg/kg) + stat IV promethazine 25 mg (0.33 mg/kg) + albuterol nebulization three times back to back of 1 mL/5 min	BP unrecordable, HR 110 BPM, O_2_ saturation 98% on high-flow oxygen
17 minutes	Third IM epinephrine dose 500 mcg (standard adult dose) + intravenous (IV) epinephrine drip (4 mcg/min)	BP from unrecordable to 60/40 mmHg, HR 126 BPM, O_2_ saturation 100% on high-flow oxygen
20 minutes	First IV epinephrine bolus 300 mcg (4 mcg/kg) + IV epinephrine drip (4 mcg/min)	BP < 60/40 mmHg 126/75 mmHg, HR 140 BPM, O_2_ saturation 100% on high-flow oxygen
24 minutes	IV epinephrine drip (4 mcg/min)	BP 88/59 mmHg, HR 110 BPM, O_2_ saturation 100% on high-flow oxygen
25 minutes	Second IV epinephrine bolus 200 mcg (2.6 mcg/kg) + IV epinephrine drip (4 mcg/min)	BP: 88/59 mmHg 140/90 mmHg, HR 125 BPM, O_2_ saturation 100% on high-flow oxygen
30 minutes	IV epinephrine drip (4 mcg/min)	BP: 132/86 mmHg, HR 93 BPM, O_2_ saturation 100% on high-flow oxygen

**Table 2:** Laboratory investigations following the anaphylactic episode.

Laboratory investigations	Result	Reference range	Laboratory investigations	Result	Reference range
White blood cells (*10^9^/L)	11.03	3.6-9.6	Troponin-I (ng/mL)	0.040	<1.5
Hemoglobin (g/dL)	17.1	12-14.5	Urea (mmol/L)	5.1	3.2-8.2
Platelets (*10^9^/L)	292	150-400	Creatinine (µmol/L)	73	53-97
Amylase (U/L)	83	30-118	Sodium (mmol/L)	139	132-146
Albumin (g/L)	49	35-52	Potassium (mmol/L)	3.7	3.5-5.5
Total bilirubin (µmol/L)	12	5-21	Chloride (mmol/L)	103	98-107
Alkaline phosphatase (U/L)	58	50-136	Bicarbonate (mmol/L)	26	24-32
Alanine aminotransferase (U/L)	16	<41	Creatine kinase (U/L)	601	35-232
G-glutamyl transferase (U/L)	28	15-85	Lactic dehydrogenase (U/L)	195	135-225

Later after stabilization of the patient’s condition, he was admitted for observation where he achieved full recovery. The patient was advised to increase fluid intake and was discharged a day later on valsartan-hydrochlorothiazide 160-12.5 mg tablet OD PO, nifedipine 30 mg tablet OD PO, metoclopramide hydrochloride 10 mg tablet TDS for 5 days, and paracetamol 500 mg tablet PRN.

## Discussion

In this case report, we discuss the successful management of a patient in severe anaphylactic shock with signs of cardiovascular compromise that has not responded to first-line management and where rescue measures were required to save the patient and prevent imminent collapse.

The patient received three medications that could act as culprits to his anaphylactic shock namely: captopril, amlodipine, and diclofenac. Angiotensin-converting enzyme inhibitors (captopril) and calcium channel blockers (amlodipine) are considered the first-line management of hypertension in certain patient demographics [4]. Diclofenac is a widely used non-steroidal anti-inflammatory drug (NSAID) in the management of pain, especially in renal colic as it has been heavily researched and has the strongest evidence of effectiveness [5]. These medications are usually safe and effective. NSAIDs have been reported to induce anaphylactic reactions, while angiotensin-converting enzyme inhibitor-induced angioedema is also well documented [6,7]. Anaphylactic reactions to amlodipine are exceedingly rare with only a few isolated events reported [8]. Due to the temporal relationship between drug administration and the onset of symptoms, the most likely offender, in this case, is diclofenac as symptoms started to develop almost immediately following its administration.

The European Academy of Allergy and Clinical Immunology (EAACI) recently updated the anaphylaxis guidelines 2021 following conducting a comprehensive systemic review of high-quality research. The agreed-upon initial first-line management of anaphylaxis is to assess the airway, breathing, circulation, disability, and exposure (ABCDE) of the patient, remove potential triggers and administer epinephrine. This is then followed by continuous monitoring, escalating to emergency teams with expertise in critical care, and providing supportive measures as needed including administering oxygen, IV fluids, nebulized epinephrine, beta-2 agonists, corticosteroids, and antihistamines [9].

Role of epinephrine in anaphylaxis

Early administration of epinephrine in anaphylaxis has been shown to reduce both rates of biphasic reactions and overall fatalities [9]. Prompt intramuscular (IM) administration of epinephrine in the mid-thigh is recommended [9]. In comparison to IM administration of epinephrine in the mid-thigh, it is found that administration of epinephrine in the deltoid or through the use of a metered-dose inhaler or subcutaneously all resulted in lower plasma levels of epinephrine [9]. IV administration of epinephrine resulted in a quicker rise in plasma levels of epinephrine but is associated with an increased risk of epinephrine overdosing and cardiovascular side effects in comparison to IM administration [9,10].

Multiple IM epinephrine injections might be required depending on the patient’s response [9]. In some special circumstances including refractory respiratory distress and hypotension, where anaphylaxis is refractory to IM epinephrine, IV epinephrine should be used [9]. Two routes to administer IV epinephrine are through an IV drip or an IV bolus. IV drips are preferred as they are controlled and can be titrated according to the patient’s response [10]. Bolus epinephrine is usually reserved for refractory cases to all the above measures [9,10]. Furthermore, the use of IV epinephrine should be restricted to healthcare professionals who are trained to use it and to a monitored clinical setting [9-11].

In this case, the patient received three IM epinephrine injections into the right deltoid (the thigh was not accessible due to extremely tight clothing and high BMI) and started on an IV drip without significant improvement. All supportive measures were also administered as a shotgun approach. Despite these interventions, the patient was still in severe grade 4 systemic anaphylactic reaction according to the Mueller grading system. The decision was made by the emergency on-call consultant to administer an IV bolus of epinephrine to prevent imminent cardiovascular collapse. This was done under close monitoring of the patient in triage level 2 and in collaboration with the intensive care unit. The patient received a total of 500 mcg, 300 mcg (3 mL stat of (1 mL of 1/10,000 epinephrine + 9 mL of NS) i.e., 4 mcg/kg) followed by a second 200 mcg (2 mL stat of (1 mL of 1/10,000 epinephrine + 9 mL of NS) i.e., 2.6 mcg/kg) administered 5 minutes apart which helped to normalize the BP.

When looking into different routes of epinephrine administration and comparing the maximum plasma concentration (Cmax) and time required to achieve Cmax (Tmax), it has been shown that IM epinephrine administration achieved the highest Cmax when compared to IV, subcutaneous, and inhaled epinephrine administration [12]. However, when comparing the Tmax required to achieve the Cmax, Cmax was achieved immediately through IV administration while Tmax was on average 32.5, 111.7 , and 45.8 minutes for IM, subcutaneous , and inhaled administration respectively [12]. Due to the patient being severely hypotensive and at risk of collapse, the delivery of epinephrine from the IM route to the target organs (heart and vascular system) is delayed. Bolus epinephrine provides a means of direct delivery to these organs hence providing the desired effect from the drug in a time-critical fashion.

The crucial role epinephrine plays in the management of anaphylaxis is owing to its pharmacological actions. Epinephrine is an endogenous catecholamine which increasingly recognized as an important metabolic hormone released in response to stress [13]. It helps mobilize energy stores and induce a number of changes in the body collectively known as the fight-or-flight response [13,14]. Through its action on alpha-1 adrenergic receptors, epinephrine administration results in increased peripheral vascular resistance, BP, and coronary artery perfusion while lessening vessel vasodilation and increased vascular permeability that occurs during anaphylaxis, which can lead to loss of intravascular fluid volume and hypotension [14]. Positive inotropic and chronotropic activity is mediated through its action on beta-1 adrenergic receptors [14]. It also increases bronchial smooth muscle relaxation and helps alleviate bronchospasm, wheezing, and dyspnea that may occur during anaphylaxis through its action on beta-2 adrenergic receptors [14]. Epinephrine also possesses vasodilator effects in the skeletal muscle which explains why IM administration is preferred over subcutaneous administration in anaphylaxis [14].

A unique aspect to consider in this case is the use of antihypertensive medications before the onset of the anaphylactic episode. Antihypertensive medications work on reducing BP through a wide range of different mechanisms. The antihypertensive effect usually starts as soon as the medication is administered with peak concentrations and maximal antihypertensive effects are achieved within 60 to 90 minutes depending on the type of antihypertensive agent [15]. Optimal control of BP is mostly achieved a few weeks after the start and titration of antihypertensive therapy [16]. Chronic use of antihypertensive medications in a few studies on animal models has been shown to downregulate adrenergic receptors which can potentially contribute to reduced efficacy of IM epinephrine [17,18]. However, our patient was not on chronic antihypertensive therapy which makes it unlikely that the antihypertensive agents played a role in the reduced response to epinephrine, but they may have played a role in the drop in BP as their antihypertensive function started to take effect along with the onset of the anaphylaxis episode.

In addition to epinephrine, high-flow oxygen should be given to patients with anaphylaxis. In those with laryngeal/pharyngeal edema, oxygen together with inhaled administration of epinephrine via a nebulizer is recommended [9]. Fluid support is crucial in patients with cardiovascular involvement to help restore circulatory volume [9]. Patients may receive inhaled beta-agonists as well as glucocorticoids to assist with bronchospasm [19,20]. Despite little evidence from the literature to support the use of these drugs specifically for anaphylaxis, it is known that beta-agonists are effective in the treatment of allergic asthma and upper airway obstruction and that steroids block arachidonic acid production, thus reducing inflammation and decreasing the likelihood of protracted anaphylaxis and biphasic reactions [19,20]. Systemic antihistamines have also been linked to reducing biphasic reactions, but the only established benefit is their role in relieving cutaneous symptoms [9,19,20].

In the critical setting, the use of these drugs in a “shotgun” approach is reasonable, but further robust evidence is required to establish their role in the management of anaphylaxis. More randomized control trials and well-designed studies would significantly add to the database as there are currently limited high-quality studies. Finally, it is important to educate the paramedical and medical staff on recognizing the signs and symptoms of anaphylactic shock and the management of typical and refractory cases according to the current best medical practice guidelines.

## Conclusions

Following evidence-based medicine is crucial in the healthcare setting. Being able to respond in a timely and effective manner to critical cases is an integral skill for any physician, especially in an emergency setting. In situations where first-line therapy is not effective, it is important to escalate management appropriately. Emergency clinicians must be able to recognize these presentations and make prompt clinical decisions. Furthermore, emergency clinicians may be faced with patients who have atypical presentations or require special consideration, such as in this case report where the patient did not respond to first-line treatments. Further research is required to establish comprehensive guidelines on the management of anaphylaxis. Currently, IM epinephrine is the mainstay treatment for anaphylaxis. IV epinephrine is not recommended as first-line but physicians with the knowledge and experience can opt to resort to it if first-line management fails. We hope this case report would add to the anaphylaxis database and demonstrate a case that has been successfully treated with IV bolus epinephrine without developing complications.
